# The cost of saving lives: Complications arising from prehospital tourniquet application

**DOI:** 10.1111/acem.15070

**Published:** 2024-12-16

**Authors:** Mor Rittblat, Sami Gendler, Nir Tsur, Irina Radomislensky, Arnona Ziv, Moran Bodas

**Affiliations:** ^1^ Israeli Defense Forces Medical Corps Tel Hashomer Ramat Gan Israel; ^2^ Department of Emergency and Disaster Management, School of Public Health, Faculty of Medical and Health Sciences Tel Aviv University Tel Aviv‐Yafo Israel; ^3^ Department of Military Medicine and "Tzameret," Faculty of Medicine The Hebrew University of Jerusalem Jerusalem Israel; ^4^ Department of Plastic and Reconstructive Surgery Hadassah Hebrew University Medical Centre Jerusalem Israel; ^5^ Department of General Surgery, Rabin Medical Center (Beilinson Campus) Tel Aviv University Petach Tiqva Israel; ^6^ Department of Otolaryngology‐Head and Neck Surgery, Rabin Medical Center Tel Aviv University Petach Tiqva Israel; ^7^ Data Science Center Gertner Institute of Epidemiology and Health Policy Research, Tel Hashomer Ramat Gan Israel

## Abstract

**Background:**

Uncontrolled hemorrhage is a leading cause of preventable death in trauma. Tourniquets (TQs) are commonly used to control bleeding in the prehospital setting, although their application is associated with risks. Therefore, this study aimed to identify complications arising from TQ use and to examine contributing risk factors.

**Methods:**

This retrospective observational study reviewed the medical records of adult trauma casualties (>18 years) hospitalized at Chaim Sheba Medical Center (SMC) between 2010 and 2020 who had a TQ applied in the prehospital setting. The primary outcome was the rate and type of complications. Logistic regression analyses identified risk factors using demographic, injury, and clinical data.

**Results:**

From 2010 to 2020, a total of 84 trauma casualties with documented prehospital TQ application were hospitalized at SMC. Of these, 20 (23.81%) experienced TQ‐related complications, including local infection, compartment syndrome, and thromboembolism. The average TQ application time was 44.2 min, with no significant difference between those with and without complications. However, significant differences were noted in the mechanism of injury (MOI), wound contamination levels, indications for TQ application, and initial blood test results (*p* < 0.05). Logistic regression analyses revealed length of stay (LOS) and injuries from falls were significantly associated with the development of complications.

**Conclusions:**

This study found that a significant trauma in prehospital settings requiring TQ application is associated with a high rate of complications. Early complications, including local infections and compartment syndrome, were more frequent, whereas late complications like thromboembolism and muscle atrophy were less common. The findings suggest that factors such as the MOI and wound contamination may contribute to these complications, yet after applying multivariate regression, LOS and falls were the only variables found to be significantly associated with the development of complications. These findings underscore the need for further research comparing casualties with and without TQ application.

## BACKGROUND

The use of tourniquets (TQs) was first recorded in the 17th century during the Battle of Besançon.^1^ Since then, the use of TQs has increased and is now adopted in various settings, including military conflicts and road traffic accidents where injuries are primarily traumatic.^2^, ^3^ Attitudes toward TQ have varied over the years. By the 21st century, TQs were considered a last resort, and at the beginning of the millennium, U.S. soldiers did not carry TQs during armed conflicts.^4^ However, today most military organizations incorporate TQ training in their combat programs,^5^, ^6^, ^7^ with prehospital civilian systems adopting a similar approach, emphasizing aggressive and early use of TQ.^8^, ^9^, ^10^

Indications for applying a TQ vary among rescue organizations, but the indisputable indications include amputation, bleeding that cannot be controlled with direct pressure, tissue mangling, and expanding subcutaneous hematoma.^11^, ^12^ Many studies have demonstrated greater survival rates among those treated with a TQ compared to those who were not.^13^, ^14^, ^15^, ^16^

While effective, the utilization of TQ is associated with notable risks, and numerous TQ‐related complications have been reported in the literature, with varying incidences.^17^, ^18^, ^19^, ^20^ Complications can manifest shortly after TQ application, ranging from hours to days, or may arise weeks later.^21^ Severe complications include conditions like compartment syndrome, which may require fasciotomy,^22^, ^23^ and ischemia–reperfusion injuries that can lead to rhabdomyolysis and subsequent acute kidney injury.^3^, ^24^ Additionally, nerve palsy and thromboembolic events such as deep vein thrombosis (DVT) have been observed and are often categorized as late or chronic complications.^22^, ^23^, ^24^, ^25^

Although there is no defined “safe time” for leaving a TQ on a bleeding limb, the literature generally recommends removing the TQ as soon as possible. Some studies suggest that leaving the TQ on for less than 2 h is associated with a lower incidence of complications,^10^, ^17^, ^26^, ^27^ whereas leaving it on for 6 h or more is strongly associated with irreversible tissue damage and limb amputation.^10^

A review of the literature reveals a lack of studies examining the relationship between prehospital treatment and in‐hospital complications following TQ application. Thus, we aim to characterize in‐hospital complications associated with TQ application and identify risk factors for their development after injury.

## METHODS

### Study design

This observational study involved a retrospective review of all casualties treated with a TQ in the prehospital setting and subsequently hospitalized between 2010 and 2020. A clinician (MR) meticulously reviewed each medical file to gather data spanning from the point of injury (POI) to in‐hospital outcomes. Complications were documented only if directly attributable to TQ application, not the initial injury. These TQ‐related complications were categorized as early or late and classified according to the Clavien‐Dindo scale.^28^ The Human Research and Ethics Committee at Chaim Sheba Medical Center (SMC; SMC‐9950‐22) and the Institutional Review Board of Tel Aviv University (0006288‐2) approved the study.

### Variables

The data collected included age, sex, evacuation platform, mechanism of injury (MOI), Injury Severity Score (ISS), length of hospitalization, and other relevant clinical and laboratory information. Wound contamination levels were also recorded with the wounds regarded clean contaminated if a minimal exposure to contaminants was seen such as the case in a clean kitchen knife cut at home, and grossly contaminated was referred to wounds exposed to heavy environmental contamination, often involving dirt, debris, or organic material such as the case in motor vehicle collision or at combat where the wound may be exposed to soil, gravel, and other contaminants.

Details about the TQ application indications and effectiveness were also noted. A TQ was considered effective if it fully stopped bleeding upon hospital arrival, and appropriateness was evaluated based on the clinical practice guidelines of Magen David Adom or the Israel Defense Forces Medical Corps,^19^, ^29^ which managed the casualty evacuations.

### Study setting and population

This study was conducted at SMC, a Level I trauma center and the largest medical center in Israel. The article adheres to the STROBE guidelines for cohort studies.^30^

The inclusion criteria aimed to identify adult casualties (>18 years) hospitalized after TQ application during prehospital treatment, either at the POI or during evacuation. Casualties who were deceased upon hospital arrival or had undocumented TQ application times were excluded. Additionally, casualties not hospitalized were excluded since in‐hospital complications data were unavailable for this population.

### Data sources

We used the Israel National Trauma Registry (INTR) to identify casualties who met the eligibility criteria. The INTR is an in‐hospital trauma registry operated by the Gertner Institute for Epidemiology and Public Health Policy Research at Tel Hashomer, Israel. The registry entries are performed by trained trauma registrars who operate the registry at 21 of 26 hospitals in Israel, including all Level I trauma centers. Data entry is supervised by trauma directors at these centers and is analyzed periodically to ensure the accuracy and completeness of the data. Notably, the database includes all hospitalized casualties or those who died in the emergency department (ED) or transferred from the ED to other hospitals but does not include those discharged from the ED or deceased prior to ED admission. Data were extracted from the Chameleon computerized clinical information system database (Version 5.17, Elad Systems).

### Data analysis

Categorical variables were compared between groups using chi‐square and Fisher's exact tests. Quantitative variables were assessed using the Mann–Whitney *U* test due to the small sample size. Categorical variables are presented as frequencies and percentages, while continuous variables are represented by medians and interquartile ranges (IQRs) or mean and standard deviation (SD). To evaluate factors associated with in‐hospital TQ‐related complications, binary logistic regression analyses were employed, assessing demographic, clinical, and treatment‐related factors hypothesized to potentially affect the complication rate of TQ application. Results are expressed as odds ratios (ORs) with corresponding 95% confidence intervals (CIs). All tests are two‐sided, and a significance level of *p* < 0.05 was considered statistically significant. All statistical analyses were conducted using SPSS software Version 27 (IBM SPSS Statistics for Windows, Version 27.0, IBM Corp., 2020).

## RESULTS

**FIGURE 1:**
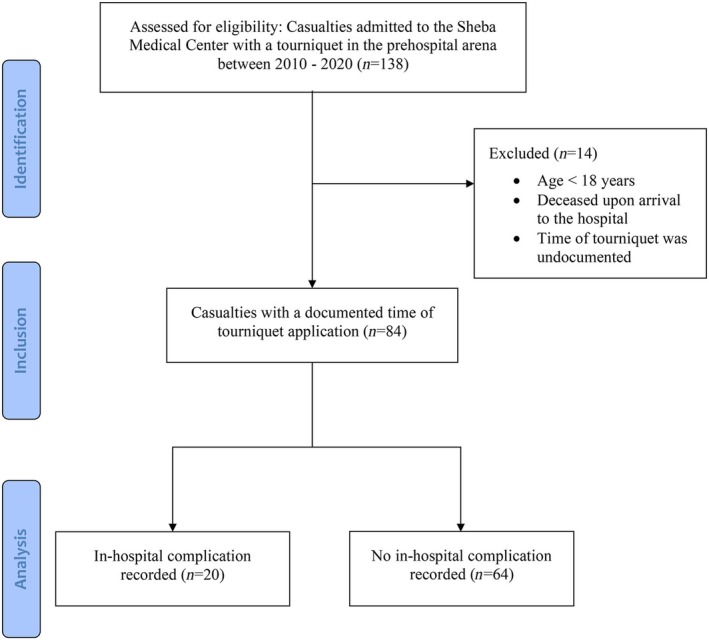
Flow diagram of the study cohort.

A flow chart of the study population is presented in Figure 1. Overall, we identified 138 casualties treated with a TQ in the prehospital setting between 2010 and 2020. Fourteen casualties were excluded due to young age (<18 years), lack of hospitalization, or unrecorded TQ time on the limb. Therefore, the study population included 84 casualties, of whom 20 (23.81%) had in‐hospital TQ‐related complications (complication group), while the remaining did not (no‐complication group).

### Casualty demographics and injury characteristics

Table 1 provides a summary of the baseline demographics and injury characteristics of the study population, stratified based on in‐hospital TQ related complications. The mean ± SD age was 38.07 ± 15.9 years, with 81 (96.4%) being male. Several statistically significant differences were observed between the study groups. The MOI in the complication group was predominantly blunt object strikes, while the noncomplication group primarily involved sharp force wounds (50% vs. 45.9%, *p* = 0.009). In addition the complication group had a 100% rate of grossly contaminated wounds compared to 71.9% in the noncomplication group (*p* = 0.005). In contrast, ISS was not statistically different between study groups, and over 90% of the study cohort had a score lower than 15. While there were no differences between the two study groups in the evacuation platform (*p* = 0.9), a statistically significant difference was found in the length of stay (LOS), with the complication group averaging 22.1 ± 14.8 days compared to the no‐complication group at 8.7 ± 8.9 days (*p* < 0.001). Regarding labs upon initial presentation, some statistically significant differences were observed, including lower hemoglobin levels in the complication group (11.4 ± 2.6 vs. 12.7 ± 2.4, *p* = 0.029) and lower creatinine (0.8 ± 0.1 vs. 1.0 ± 0.3, *p* = 0.007), among others (for further details, refer to Table 1).

**TABLE 1:** Basic and injury casualty's characteristics.

	In‐hospital complications (*n* = 20, 23.81%)		No in‐hospital complications (*n* = 64, 76.19%)		Overall (*n* = 84)		*p*‐value^a^
Age (years)	36.0 ± 13.6		38.7 ± 16.6		38.07 ± 15.9		0.6
Male sex	20	(100.0)	61	(95.3)	81	(96.4)	0.4
Preexisting medical condition	3	(15.0)	15	(23.4)	18	(21.4)	0.5
Evacuation platform							
Ambulance w/medics only	11	(55.0)	34	(53.1)	45	(53.6)	0.9
Ambulance w/ALS	7	(35.0)	25	(39.1)	32	(38.1)	
Airborne (w/ALS)	2	(10.0)	5	(7.8)	7	(8.3)	
MOI							
Sharp force	2	(10)	29	(45.3)	31	(36.9)	0.009*
Blunt object strike	10	(50)	11	(17.2)	21	(25.0)	
Motor vehicle collisions	4	(20)	14	(21.9)	18	(21.4)	
Explosion	3	(15)	6	(9.4)	9	(10.7)	
Falls	1	(5)	4	(6.3)	5	(6.0)	
Wounds contamination levels							
Clean contaminated	0	(0)	18	(28.1)	18	(21.4)	0.005*
Grossly contaminated	20	(100)	46	(71.9)	66	(78.6)	
ISS							
1–8	6	(30.0)	28	(43.8)	34	40.5)	0.33
9–14	12	(60.0)	31	(48.4)	43	(51.2)	
16–24	2	(10.0)	2	(3.1)	4	(4.8)	
25+	0	(0)	3	(4.7)	3	(3.6)	
LOS (days)	22.1 ± 14.8		8.7 ± 8.9		11.9 ± 11.9		<0.001*
ICU hospitalization length (days)	0.2 ± 0.7		0.3 ± 1.3		0.2 ± 1.2		0.977
First blood tests at hospital admission							
Hgb	11.4 ± 2.6		12.7 ± 2.4		12.4 ± 2.5		0.029*
WBC	11.0 ± 5.1		10.7 ± 4.3		10.8 ± 4.5		0.9
Sodium (Na^+^)	137.6 ± 2.1		138.9 ± 2.4		138.6 ± 2.4		0.03*
Potassium (K^+^)	4.2 ± 0.6		4.1 ± 0.4		4.1 ± 0.5		0.7
Chloride (Cl^−^)	105.5 ± 2.9		105.8 ± 5.6		105.7 ± 5.0		0.7
LDH	242.0 ± 90.1		229.7 ± 84.0		232.8 ± 85.2		0.7
Uric acid	4.9 ± 1.2		5.0 ± 1.4		5.0 ± 1.3		>0.9
Creatinine	0.8 ± 0.1		1.0 ± 0.3		0.9 ± 0.3		0.007*
Urea	29.9 ± 6.1		34.9 ± 15.8		33.7 ± 14.2		0.4
AST	40.5 ± 17.5		36.7 ± 36.4		37.6 ± 32.8		0.037*
ALT	27.3 ± 17.5		25.0 ± 22.5		25.5 ± 21.3		0.3
CPK	365.0 ± 168.4		1459.8 ± 3343.8		1260.8 ± 3040.2		0.4
CRP	3.1 ± 3.8		17.3 ± 36.1		14.8 ± 33.2		0.4

*Note*: Data are reported as mean ± SD or *n* (%).

Abbreviations: ALS, advanced life saver; ALT, alanine transaminase; AST, aspartate aminotransferase; CPK, creatinine phosphokinase; CRP, C‐reactive protein; Hgb, hemoglobin; ICU, intensive care unit; ISS, Injury Severity Score; LDH, lactic acid dehydrogenase; LOS, length of stay.

^a^ *p*‐values acquired from Fisher's exact test or Mann–Whitney *U*‐test.

* The numbers are statistically significance.

### TQ application features

Table 2 summarizes the TQ features of the study groups. There were no differences in the mean duration of the TQ application, with a mean time of 40.8 ± 21.8 min for the complication group versus 45.3 ± 46.4 min for the noncomplication group. In the study cohort, 96.4% of TQ application were effective, with no differences between study groups. No differences were also observed in the appropriateness of the TQ application, with 17/20 (85.0%) applications being appropriate in the complication group versus 49/64 (76.6%) in the noncomplication group.

**TABLE 2:** TQ application features.

	In‐hospital complication (*n* = 20, 23.81%)		No in‐hospital complication (*n* = 64, 76.19%)		Overall (*n* = 84)		*p*‐value^a^
Duration of the TQ application (min)	40.8 ± 21.8		45.3 ± 46.4		44.2 ± 41.8		0.5
TQ was applied effectively	20	(100)	61	(95.3)	81	(96.4)	0.4
TQ was applied appropriately	17	(85.0)	49	(76.6)	66	(78.57)	0.5
TQ application indications							
Massive hemorrhage	2	(10.0)	26	(40.6)	28	(33.3)	0.023*
Traumatic amputation	9	(45.0)	13	(20.3)	22	(26.2)	
Contraindicated	3	(15.0)	15	(23.4)	18	(21.4)	
Multiple injuries in the same limb	4	(20.0)	7	(10.9)	11	(13.1)	
Penetrating fragment injury	2	(10)	3	(4.7)	5	(6)	

*Note*: Data are reported as mean ± SD or *n* (%).

Abbreviation: TQ, tourniquet.

^a^ *p*‐values acquired from Fisher's exact test or Mann–Whitney *U*‐test.

* The numbers are statistically significance.

In contrast, a statistically significant difference was observed between the study groups regarding the indications for TQ application (*p* = 0.023). In the complication group, the leading indication was traumatic amputation (nine of 20 cases, 45%), followed by multiple injuries in the same limb (four of 20 cases, 20%). In the noncomplication group, the primary indication was massive hemorrhage (26 of 64 cases, 40.6%), followed by traumatic amputation (13 of 64 cases, 20.3%).

**FIGURE 2:**
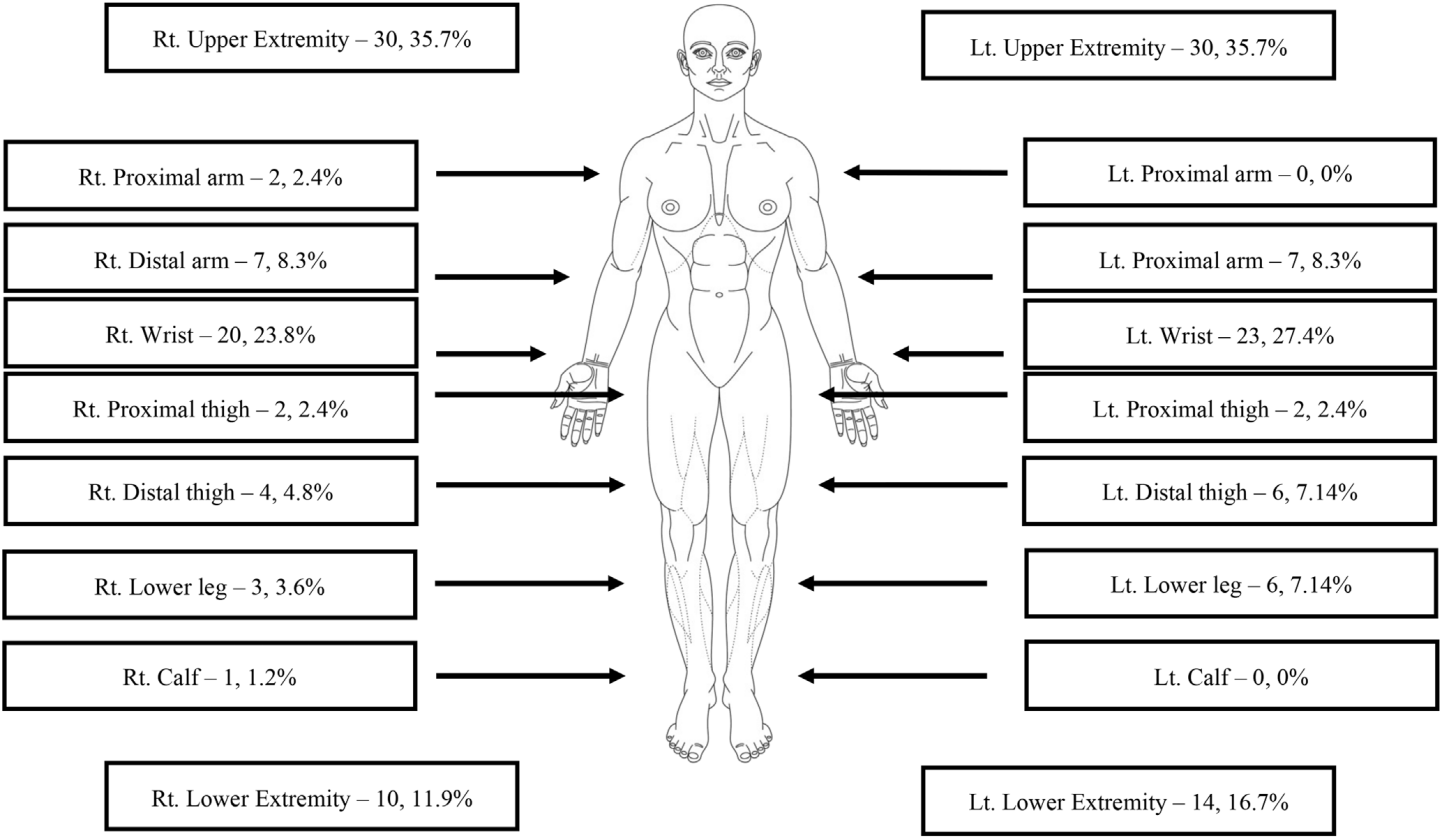
Body diagram displaying the anatomical area and proportions of the TQ's application of the study cohort. TQ, tourniquet.

Figure 2 displays the anatomical application of the TQ in the cohort. Regardless of the side, 71.4% of TQ were applied on the upper extremities, with half (43/84) positioned at wrist height. There were no statistically significant differences between the study groups in the distribution of the limb treated with a TQ, nor in the anatomical area of the TQ application (*p* > 0.05).

### TQ complication nature

**FIGURE 3:**
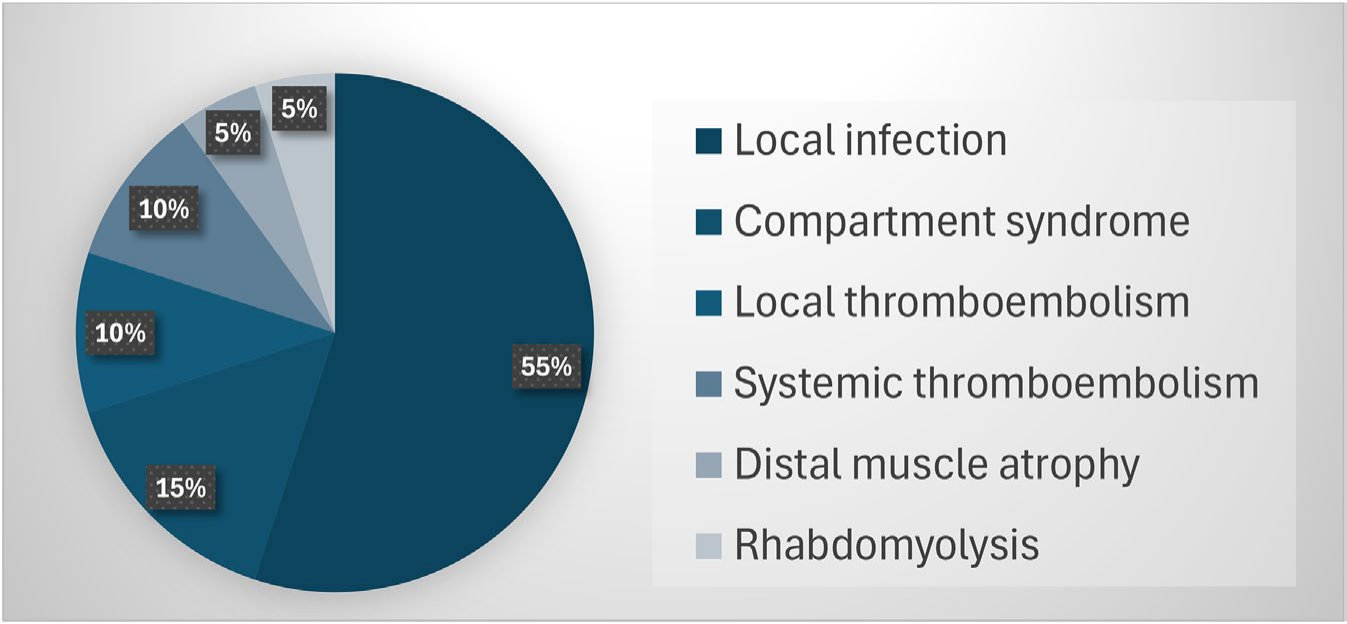
TQ complication nature. TQ, tourniquet.

Figure 3 describes the TQ‐related complications documented following the TQ application. The most common complication recorded was local infection, with nine cases (55%; 10.7% of all casualties), followed by three cases (15%; 3.6% of all) of compartment syndrome, two cases (10%; 2.4% of all) each of local and systemic thromboembolism events, and an additional two cases (10%; 2.4% of all), one each (5%; 1.2% of all) of distal muscle atrophy and rhabdomyolysis. In all cases where amputations were recorded, they were related to the injury mechanism rather than the TQ application. Early TQ‐related complications that developed within hours to days from the TQ application were twice as more prevalent with 13 cases (65%; 15.5% of all) versus seven cases (35%; 8.33% of all) of the late complication.

In accordance with the Clavien‐Dindo classification, 17/20 (85%; 20.2% of all) of the complications were rated as Clavien I, one (5%; 1.2% of all) as Clavien IIIb, and two (10.0%; 2.4% of all) as Clavien IV. Specifically, the two cases of pulmonary embolism (PE), representing systemic thromboembolic events, were classified as Clavien Grade IV, while one case of compartment syndrome requiring fasciotomy was classified as Clavien Grade IIIb. All remaining complications were classified as Clavien Grade I.

### Multivariable logistic regression of TQs complication

**FIGURE 4:**
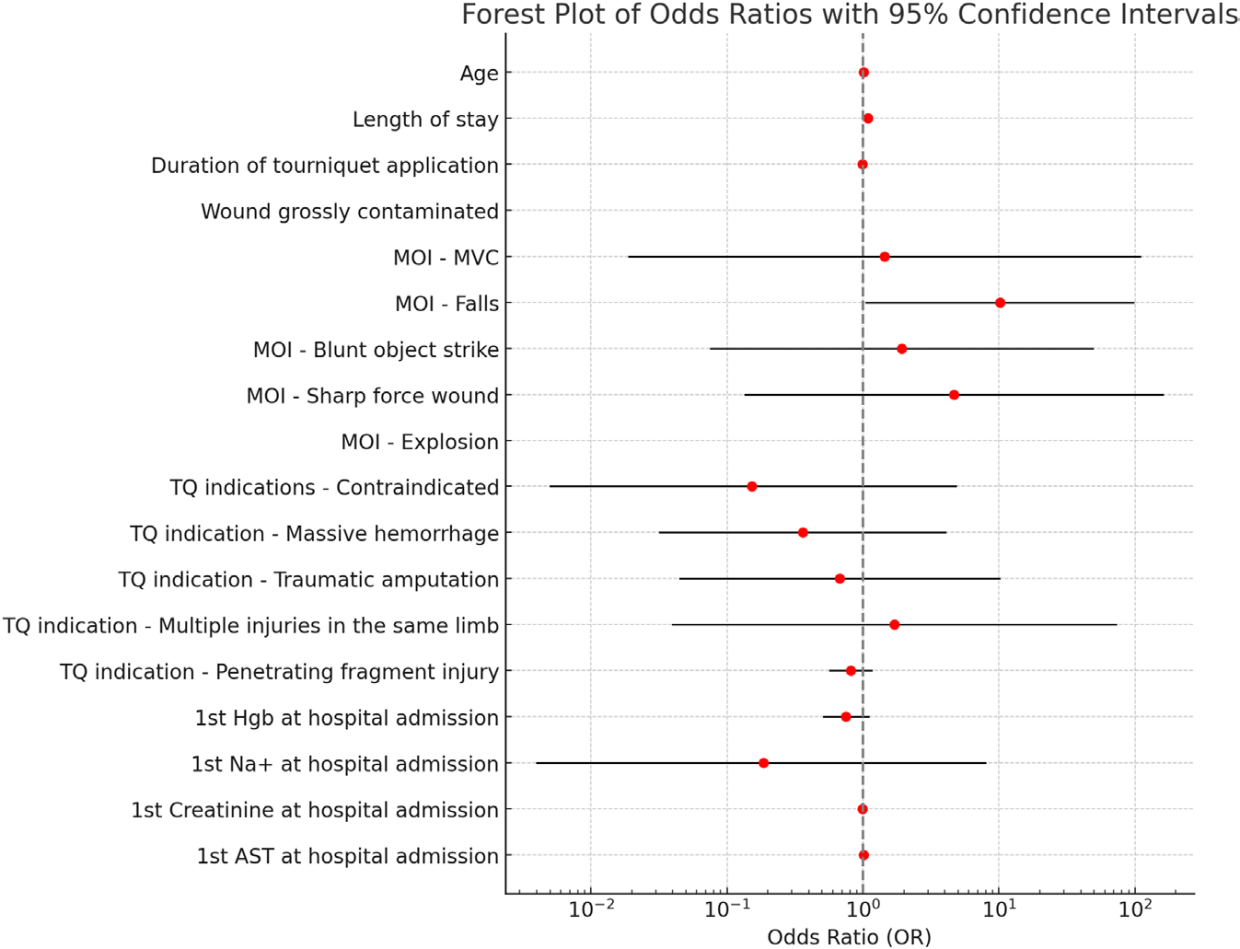
Complications after TQ applications predictors. Forest plots with a logarithmic x‐axis of forward stepwise multivariate logistic regression analysis for complications after TQ application. Red dots represent the ORs with 95% CIs for each variable. Black dashed line marks the “no effect” line (OR 1), serving as a reference for interpretation. AST, aspartate aminotransferase; Hgb, hemoglobin; MOI, mechanism of injury; MVC, motor vehicle collisions; TQ, tourniquet.

Following the univariable regression analysis, we conducted a forward stepwise multivariate linear regression analysis on the entire cohort to identify the major statistically significant predictors for in‐hospital complications following the application of a TQ in the prehospital setting. The analysis revealed two variables significantly associated with in‐hospital complications: LOS (OR 1.086, 95% CI 1.014–1.162, *p* = 0.018) and injuries due to fall (OR 10.141, 95% CI 1.054–97.622, *p* = 0.045; see Figure 4). Age, duration of the TQ application, TQ application indication, initial blood test results on admission, and other the remaining MOIs were not significantly associated with increased or decreased odds of complication (refer to Table S1 for complete results).

## DISCUSSION

This study examined the rate of in‐hospital complications related to TQ use among casualties who had a TQ applied in the prehospital setting and were subsequently evacuated to a single Level I trauma center. A high complication rate (23.81%) was observed despite the relatively short mean TQ application time (44.2 min). The most common TQ‐related complication was local infection, occurring in nine out of 20 cases (55%). The development of complications was associated with several risk factors, including wound contamination levels. Decreased hemoglobin levels, reduced sodium levels, and low creatinine levels at admission. Additionally, the complication group had a longer LOS and predominantly sustained blunt object strikes. However, in the regression model, only LOS and injuries due to falls were significantly associated with increased odds of developing complications following TQ application.

TQs, like other medical technologies, carry risks; however, not using them may lead to increased blood loss and worse outcomes.^13^, ^14^ Bonk et al.^31^ found that the mortality rate more than doubled when TQs were applied in the ED rather than in the prehospital setting. To date, the consensus on the safe period for leaving a TQ on is generally considered to be 2 h,^10^, ^17^, ^26^, ^27^, ^32^ with studies reporting a wide range of TQ‐related complication rates, from none to up to a third of cases.^3^, ^17^, ^18^, ^19^, ^20^, ^26^, ^32^, ^33^ In this study, we report a high TQ‐related complication rate despite the short mean application time of 44.2 min, which is well below the “safe time” of 2 h. This finding is surprising, especially since the overall injury burden was not severe, as indicated by the median ISS. These injuries often necessitated the application of a TQ and were typically life‐threatening, requiring immediate, intensive medical attention to address severe body injuries, including vascular damage. Another explanation could be the data collection process, which involved meticulously reviewing each medical record by hand and conducting a thorough investigation into any documented complications. This method ensured a high degree of certainty that the TQ‐related complications were accurately attributed to the TQ application rather than arising from the injury mechanism or hospitalization itself. To date, few studies have employed this more accurate data‐gathering method.

Despite the limited cohort size, six distinct TQ‐related complications were identified through manual review of each medical file within the study population. Notably, the most prevalent early complication was local infection, occurring in 13.1% of cases (11/84), which is higher than the incidence rates reported in other studies, where rates are typically in the single digits.^23^, ^24^ Although other studies did not specify wound contamination levels, the higher infection rate in our study may be attributed to the high percentage of grossly contaminated wounds, with an overall rate of 78.6%, and 100% contamination among the complication group. All infection cases were classified as Clavien Grade I, as they were managed with a short course of antibiotics, resulting in resolution within days, consistent with expectations for superficial wound infections. The high rate of grossly contaminated wounds among patients who developed complications (100%) aligns with findings in trauma literature, where contamination is a key predictor of local infection and compartment syndrome.^34^ This suggests that the observed complications may primarily result from the nature of the injuries rather than TQ use alone.

Another early complication observed was compartment syndrome, which occurred in three cases (3.57%). Of these, two were classified as Clavien Grade I due to conservative management, while one case (1.2%) was classified as Clavien Grade IIIb because it required a fasciotomy. The patient who underwent fasciotomy had a TQ applied for 60 min before hospital arrival and sustained a partial amputation injury to the distal femur.

The association between blunt trauma and a higher rate of complications, including compartment syndrome, aligns with established clinical observations.^34^, ^35^ Blunt injuries are known to cause extensive soft tissue damage, leading to swelling and increased compartment pressures, which may necessitate fasciotomy.^35^ These mechanisms likely explain the significantly higher complication rate observed in blunt trauma compared to penetrating injuries in our cohort. Additionally, when comparing our incidence rate to other studies, our findings align with those of Scerbo et al.,^22^ who reported a low fasciotomy incidence of 2%. However, their study did not specify the MOI or TQ duration.

The late complications group included only five cases, four of which were thromboembolic events. Among these, two cases were diagnosed with DVT in the limb where the TQ was applied, and two presented with PE, both classified as Clavien Grade IV due to their life‐threatening nature. Both PE cases involved generally healthy young males with lower‐extremity injuries. One patient sustained a blast injury with multiple fragments and had a TQ applied for 30 min, while the other underwent TQ application for 15 min due to a traumatic amputation from blunt trauma. Both were taken to the operating room shortly after ED admission and were diagnosed with PE during hospitalization. All four thromboembolic cases were successfully treated with systemic anticoagulation, with no need for angiography or surgical intervention. In comparison, Scerbo et al.^22^ reported a 9% incidence of DVT following TQ application, which is slightly higher than the 4.8% observed in our cohort. Additionally, a single case (1%) of nerve palsy was identified, attributed to TQ application rather than the initial injury, as indicated by physical examination findings. This incidence aligns with findings by Kue et al.^25^ who also reported a single case (1%) of nerve palsy. The incidence of other complications was below 5% of the entire cohort (one or two cases), a rate comparable to those observed in other studies with similarly small sample sizes.^22^, ^23^

Given the retrospective nature of our study, it was challenging to determine whether the late complications—including thromboembolic events such as PE and the case of nerve palsy—were solely attributable to TQ use, partially due to subsequent surgical interventions, or related to the injuries themselves. Similar concerns have been raised in other studies,^22^, ^24^, ^25^ underscoring the need for further research, preferably through prospective validation studies.

Interestingly, no amputations due to the TQ application were recorded in the study sample. Several reasons may explain this finding. First, the short TQ application time in the study cohort. Shorter application times for TQ are widely recognized in medical research as being associated with a reduced risk of amputation, as prolonged application can lead to significant tissue damage and complications that may necessitate amputation.^17^ Second, the capabilities of the trauma center, as a Level I national referral center with an in‐house shock trauma unit and round‐the‐clock availability of microsurgery, likely played a role.

In this study, no statistically significant differences were found between the study groups in the effectiveness, appropriateness, or anatomical location of the TQ application. Additionally, no correlation was found between these variables and the TQ complication rate. Our results are consistent with other studies that have examined the effectiveness and appropriateness of TQ application and its relation to complications, finding no correlation between the two.^22^, ^24^, ^36^ Moreover, although the proportions of TQ application indications differed between the study groups, these differences did not demonstrate statistical significance in the regression model. As far as the location of the TQ application, our results show that the majority of TQ (71.4%) were applied to the upper limbs, as shown in other papers.^22^, ^23^, ^37^, ^38^

Comparing the laboratory results at hospital admission revealed statistically significantly lower hemoglobin counts, diminished sodium levels, worsened liver function, and slightly better kidney function (*p* < 0.05) in the complication group. The increased probability of major bleeding in the complication group may explain the lower hemoglobin count. The relationship between low hemoglobin count and increased mortality has been thoroughly investigated in trauma settings, with mortality rates increasing exponentially as hemoglobin values decrease.^22^, ^39^, ^40^ Concerning sodium values and creatinine levels, little is known about the relationship between these factors and trauma. However, abnormal sodium levels and acute kidney injury have been associated with increased complications and mortality in hospitalized casualty.^41^ A study by Inaba et al.^42^ reported 2% incidence of renal failure (two of 87 cases) after TQ application, while Scerbo et al. reported a 3% (three of 105 cases) renal failure incidence.^22^ However, the casualties suffered from the condition during hospitalization, and a direct link between the TQ application and the apparent complication was not proven. Nonetheless, after applying a forward stepwise multivariate linear regression analysis, the laboratory findings did not remain significant, and only LOS (OR 1.086, 95% CI 1.014–1.162, *p* = 0.018) and injuries due to fall (OR 10.141, 95% CI 1.054–97.622, *p* = 0.045) remained statistically significant.

There is a notable paucity of data in the literature regarding the direct complications following TQ application after traumatic injury, with few studies specifically addressing this issue. In particular, information on laboratory findings obtained shortly after injury and TQ application is limited. The blood test results in our study reflect the medical condition of casualties shortly after TQ application, conducted in the shock room soon after ED admission and prior to any hospital interventions. This suggests that hemoglobin levels, along with kidney and liver function, may be directly associated with the development of complications and ischemia in the affected limb. Yet, due to the retrospective design of this study, the potential for complications being partially attributable to the injury mechanism or hospitalization process cannot be fully excluded. Furthermore, without a comparison group of patients who did not receive TQ application, it is challenging to determine whether the observed complications are directly attributable to TQ use or reflect the severity and nature of injuries that necessitate TQ application. Therefore, additional research involving matched comparison groups, larger cohorts, and prospective study designs is required to accurately identify the nature and incidence of these complications and to discover additional factors associated with in‐hospital complications following TQ application.

## LIMITATIONS

There are several limitations to this study. First, its retrospective nature introduces potential reporting bias and data omissions inherent to the data source. Nonetheless, the data were originated from the medical sheets who were manually reviewed to determine the nature of the complications and their relevance to TQ application. Second, the retrospective design of the study, combined with the surgical procedures many casualties underwent shortly after admission, and the high prevalence of gross wound contamination made it difficult to determine whether the complications were exclusively due to TQ use. Third, this study did not include a comparison group of trauma patients who did not receive TQ application, which limits the ability to isolate the impact of TQ on the observed complications. Fourth, the study cohort may be skewed toward critically injured casualties admitted to the hospital, which may have led to an underrepresentation of the complication rate. Fifth, the study sample size is small and includes a single Level I trauma center. Further research should be conducted on a larger scale with multi‐institutional analysis.

## CONCLUSIONS

The findings demonstrate that tourniquet application in the prehospital setting carries risks, and even with a short mean application time, the complication rate remains high. The number of hospitalization days was the only variable associated with greater odds of complication development after a tourniquet application. Yet, while the association between tourniquet use and complications is noteworthy, the absence of a comparison group limits the ability to attribute these complications solely to tourniquet application. The findings suggest that complications may also be influenced by Injury Severity Score, wound contamination, and other patient‐specific factors. Moreover, It may be hypothesized that given a larger sample size, the hemoglobin levels, and liver functions, could be associated with the development of complications and ischemia in the affected limb. Therefore, further studies including matched analyses of patients with and without tourniquet application are needed to clarify and delineate the specific role of tourniquet application following significant trauma in the prehospital setting.

## AUTHOR CONTRIBUTIONS

Mor Rittblat: study concept and design, analysis and interpretation of the data, drafting of the manuscript. Sami Gendler: drafting of the manuscript, critical revision of the manuscript for important intellectual content. Nir Tsur: drafting of the manuscript, critical revision of the manuscript for important intellectual content. Irina Radomislensky: study concept and design, acquisition of the data, analysis and interpretation of the data. Arnona Ziv: acquisition of the data, analysis and interpretation of the data. Moran Bodas: study concept and design, acquisition of the data, analysis and interpretation of the data, critical revision of the manuscript for important intellectual content, statistical expertise.

## CONFLICT OF INTEREST STATEMENT

The authors declare no conflicts of interest.

## Supporting information


**Table S1.** Tourniquet application categorized by anatomical area.

**Table S2.** Forward stepwise multivariate linear regression model along with the statistical values of it.

## Data Availability

Full demographic data pertaining to individuals cannot be disclosed to ensure subjects’ anonymity and data security policies in the SMC. Derived data supporting the findings of this study may be made available from the corresponding author MR upon request.
