# Supplementary material for: The cost of saving lives: Complications arising from prehospital tourniquet application
Source: Acad Emerg Med. 2024 Dec 16;32(5):532–41. doi: 10.1111/acem.15070 (PMC12077063; doi:10.1111/acem.15070)
Supplement: Supplementary file 1 — Table S1. Tourniquet application categorized by anatomical area. [file ACEM-32-532-s001.pdf]

Table 1s – Tourniquet application categorized by anatomical area

	In-hospital complication, n=20, 23.81%		No In-hospital complication, n=64, 76.19%		Overall n=84		p-value [a]
Limb with TQ application - N (%)							
Right lower extremity	3	15.0%	7	10.9%	10	11.9%	0.4
Left lower extremity	6	30.0%	8	12.5%	14	16.7%	0.07
Right upper extremity	7	23.3%	23	35.9%	30	35.7%	>0.9
Left upper extremity	4	20.0%	26	40.6%	30	35.7%	0.09
**Anatomical area of TQ application – N (%)**							
Calf	0	0.0%	1	1.6%	1	1.2%	
Lower leg	2	10.0%	7	10.9%	9	10.7%	
Distal thigh	5	25.0%	6	9.4%	11	13.1%	0.4
Proximal thigh	2	10.0%	2	3.1%	4	4.8%	
Wrist	9	45.0%	34	53.1%	43	51.2%	
Distal arm	2	10.0%	12	18.8%	14	16.7%	
Proximal arm	0	0.0%	2	3.1%	2	2.4%	

a p-values acquired from Fisher's Exact Test or Mann-Whitney U test.

Abbreviations: TQ – Tourniquet, Std.D – Standard deviation.
