# Supplementary material for: The cost of saving lives: Complications arising from prehospital tourniquet application
Source: Acad Emerg Med. 2024 Dec 16;32(5):532–41. doi: 10.1111/acem.15070 (PMC12077063; doi:10.1111/acem.15070)
Supplement: Supplementary file 2 — Table S2. Forward stepwise multivariate linear regression model along with the statistical values of it. [file ACEM-32-532-s002.pdf]

Table 2s – Forward stepwise multivariate linear regression model

Variable	B (Coefficient)	S.E	Wald Chi-Square	p - value	95% CI for OR	
					Lower	Upper
Age	0.011	0.032	0.126	0.723	0.95	1.077
LOS	0.082	0.035	5.588	**0.018**	1.014	1.162
Duration of tourniquet application	-0.012	0.011	1.256	0.262	0.966	1.009
Wound grossly contaminated	18.336	8967.827	0	0.998	0	.
4.7250.317MOI - MVC	0.363	2.207	0.027	0.87	0.019	108.566
MOI - Falls	2.317	1.155	4.02	**0.045**	1.054	97.622
MOI - Blunt object strike	0.658	1.647	0.16	0.689	0.076	48.756
MOI - Sharp force wound	1.542	1.803	0.731	0.392	0.136	160.135
MOI - Explosion			1.832	0.767		
TQ indications - Contraindicated	-1.877	1.76	1.137	0.286	0.005	4.821
TQ indication - Massive hemorrhage	-1.023	1.231	0.691	0.406	0.032	4.013
TQ indication - Traumatic amputation	-0.397	1.38	0.083	0.774	0.045	10.049
TQ indication -Multiple injuries in the same limb	0.532	1.913	0.077	0.781	0.04	72.403
TQ indication - Penetrating fragment injury	-0.207	0.182	1.292	0.256	0.569	1.162
1st Hgb at hospital admission	-0.289	0.193	2.236	0.135	0.513	1.094
1st Na^+ at hospital admission	-1.688	1.915	0.777	0.378	0.004	7.894
1st Creatinine at hospital admission	-0.008	0.016	0.253	0.615	0.96	1.024
1st AST at hospital admission	0.011	0.032	0.126	0.723	0.95	1.077

- LOS – Length of stay, MOI - Mechanism of injury, MVC - Motor vehicle collisions, TQ – Tourniquet, Hgb - Hemoglobin, AST - Aspartate Aminotransferase, S.E: Standard Error, OR: Odds Ratio, CI: Confidence Interval.

- B (Coefficient): The log odds that the predictor variable will be present (a positive coefficient indicates an increase in odds, while a negative coefficient indicates a decrease).
- Standard Error: The standard error of the coefficient estimate.
- Wald Chi-Square: The chi-square statistic to test the null hypothesis that the coefficient (beta) is equal to zero.
- P-Value: The probability of obtaining the observed Wald Chi-Square statistic (or a more extreme value) under the null hypothesis.
- Odds Ratio (OR): The exponentiation of the B coefficient, providing the change in odds for a one-unit change in the predictor variable.
